# Microbial Decontamination of Fresh-Cut Carrots via Cold Atmospheric Plasma Treatment: Effect on Physicochemical and Nutritional Properties During Storage

**DOI:** 10.3390/foods14091599

**Published:** 2025-05-01

**Authors:** Efe Bakla, Ufuk Bağcı

**Affiliations:** 1 Edirne Food Control Laboratory Directorate, 22030 Edirne, Turkey; efebakla@trakya.edu.tr; 2 Department of Food Engineering, Trakya University, 22180 Edirne, Turkey

**Keywords:** cold atmospheric plasma, fresh produce, microbial inactivation, surface decontamination, nonthermal processing

## Abstract

The extension of shelf-life and enhancement of the safety and quality of fresh-cut ready-to-eat vegetables is an ongoing public health concern. The present study investigated the efficacy of cold atmospheric plasma (CAP) treatment for the decontamination of fresh-cut carrots inoculated with *Escherichia coli*. An atmospheric plasma jet system operating at 1 kVA was utilized for treatment with varying plasma jet nozzle to sample distances (10–40 mm), exposure times (10–60 s) and either argon or dry air at 3 bar as working gases. It was demonstrated that both working gases achieved more than 4 log reductions in *E. coli* within 60 s of treatment while maintaining carrot surface temperatures below 50 °C. During 3-week storage at 4 °C, the immediate effects of plasma treatment on quality parameters were found to be minimal, with no significant changes observed in color (Δ*E* < 3.0) parameters, β-carotene content, ascorbic acid levels, total phenolic content (TPC), or total antioxidant activity (TAA) following either treatment. Additionally, plasma-treated carrots retained their firmness, showing no significant texture loss, whereas untreated controls experienced a firmness decline of approximately 9% by the end of storage. Notably, TPC increased by up to 41%, and TAA increased significantly (*p* < 0.05) in plasma-treated samples during storage, especially in dry air plasma-treated carrots. These results demonstrated that CAP treatment can be successfully applied for rapid inactivation of *E. coli* on fresh-cut carrot surfaces while preserving original quality characteristics during refrigerated storage, offering potential as non-thermal preservation technology for fresh produce.

## 1. Introduction

Over the years, a wide variety of minimally processed fruits and vegetables have been produced to address the growing consumer demand for fresh-like and easy-to-prepare products, in line with the increasing trend towards healthier lifestyles [1]. Carrot (*Daucus carota* L.) is one of the most popular vegetables in fresh-cut form (sliced, diced or shredded) due to its versatility and high content of health-promoting phytonutrients such as carotenoids, ascorbic acid, and phenolic compounds [2]. Among these, β-carotene is particularly important, both nutritionally and commercially. As a precursor of vitamin A, β-carotene plays a crucial role in supporting immune function, maintaining visual health, and protecting cells against oxidative damage [3,4].

Despite their nutritional benefits, fresh-cut produce are prone to rapid quality deterioration following processing. Mechanical operations such as peeling and cutting disrupt plant cell structures, thereby accelerating metabolic activities like respiration and ethylene production, which in turn increase susceptibility to microbial growth and spoilage [5]. Although such issues (wounding, discoloration, etc.) are common across most fresh-cut produce, carrots are especially vulnerable to surface whitening (commonly known as “white blush”) and the loss of their characteristic bright orange color during storage. Carrots with higher water content and softer texture are more susceptible to microbial invasion, as they offer a favorable environment for the growth of pathogens such as *Erwinia* and *Geotrichum* [6]. They also tend to lose their firmness and develop off-odors over time, which negatively impacts their physical and nutritional quality [7]. Furthermore, unless appropriate hygienic practices are strictly followed, the risk of foodborne outbreaks linked to microbial contamination increases significantly. In fact, the number of foodborne illness outbreaks associated with minimally processed fruits and vegetables has increased dramatically in the past decades [8]. Several of these outbreaks have been associated with carrots [9,10], the majority of which were caused by pathogenic *Escherichia coli* strains, *Yersinia*, *Shigella* and *Salmonella* spp.

Primary postharvest sanitizing treatments for decontamination of food-borne pathogens used at the industrial level involve the use of chemical agents such as chlorine solutions, which can leave harmful by-products with potential health concerns and environmental issues [11]. Moreover, the effect of chlorine-based treatments is limited and typically results in <2 log units of pathogenic microflora, losing their effectiveness by reacting with organic constituents in foods [12]. In recent years, CAP technology has gained significant attention as an innovative non-thermal food decontamination method. CAP involves the ionization of gas molecules through electrical discharge at atmospheric pressure, resulting in a complex mix of reactive oxygen and nitrogen species (ROS/RNS), UV photons, and charged particles [13,14]. These agents collectively exert antimicrobial effects by damaging microbial cell walls, membranes, and genetic material [15,16]. These species do not leave any residual trace contaminants behind due to their very short lifetimes [17].

The effectiveness of CAP treatments can vary significantly depending on process parameters such as exposure time, working gas composition, gas flow rate, power intensity, and the distance between the plasma source and the food surface. Noble gases like argon are known to produce more stable and homogeneous plasma, while air or oxygen-rich mixtures enhance oxidative effects. However, excessive exposure or high-energy treatment can also damage food tissues or alter nutritional compounds [18,19]. The surface characteristics and chemical composition of the food, as well as the type of microorganism, also play a vital role in the decontamination efficiency of plasma [19].

Several recent studies have confirmed the antimicrobial potential of CAP against fresh produce. For instance, a reduction of 2.1 log CFU g^−1^ in the native microflora of fresh-cut carrot disks sealed in plastic packaging was achieved using dielectric barrier discharge (DBD) plasma [20]. Similarly, Silvetti, et al. [21] showed that atmospheric pressure plasma jet treatment effectively reduced microbial load on fresh-cut lettuce by 1.3 log CFU g^−1^ within 15 s while extending shelf life by 3–4 days without adversely affecting physicochemical properties. However, information on possible alterations in physicochemical and nutritional characteristics that might occur in the product due to the interaction of reactive species with the food components is still limited.

In this study, a CAP jet was utilized for surface decontamination of fresh-cut carrots. The effect of working gas on plasma efficacy was evaluated comparatively with respect to *E. coli* inactivation, as well as the physicochemical and nutritional characteristics of the fresh-cut carrots. In this context, in the first part of the study, various plasma conditions (jet nozzle to substrate distance and exposure time) were evaluated for each working gas (argon and dry air) to achieve maximum surface decontamination efficacy on *E. coli*-inoculated carrot samples. In the second part, a comparative evaluation of both the microbiological and the physicochemical quality characteristics of fresh-cut carrots treated under selected conditions was conducted during a three-week refrigerated storage period.

## 2. Materials and Methods

### 2.1. Materials

All media used in this study were purchased from Merck (Darmstadt, Germany). High-performance liquid chromatography (HPLC) or analytical-grade chemicals were purchased from Sigma-Aldrich (Steinheim, Germany).

### 2.2. Raw Material, Handling and Storage

Fresh whole carrots (*Daucus carota* L.) measuring approximately 150–200 mm in length and 32–35 mm in width and with no visible damage and uniform color were supplied from a local market in Edirne, Turkey. Carrots were hand-sorted to select undamaged ones and stored at 4 °C overnight before use. All carrots were thoroughly washed using deionized water to remove surface dirt.

### 2.3. Sample Inoculation

For surface sterilization before inoculation, carrots were dipped into 70% ethanol for 1 min and dried. Subsequently, carrots were hand-peeled using sterilized peeling tools to obtain a uniform diameter of about 30 mm. For the inoculation of carrots, the stock culture of *E. coli* ATCC 25922 was activated in tryptic soy broth at 37 °C for 24 h before the preparation of the inoculum. Cells in the subculture were separated by centrifugation (10,000× *g* for 10 min) (3 K30, Sigma, Osterode am Harz, Germany) at 4 °C. Pellets were washed and centrifuged twice in sterile physiological saline. Cells were resuspended in physiological saline to obtain approximately 9 log CFU mL^−1^.

Carrots were then sprayed with a spray bottle (0309-1100, Bürkle, Bad Bellingen, Germany) for inoculation. Each spray stroke was about 1.2 mL, and every carrot sample was sprayed on both sides for even distribution (top and bottom) ten times. After inoculation, samples were drained for 1 h under refrigeration to remove excess inoculum before plasma treatment.

### 2.4. Sample Preparation for Plasma Treatment

Preliminary analyses indicated that the nutritional composition of carrots varies significantly from top end to bottom end. However, no significant differences (*p* < 0.05) were observed when carrots were longitudinally halved, suggesting a uniform composition along the vertical axis for each part. Therefore, a standardized sample preparation method was adopted to improve the accuracy of quality assessments.

After discarding the lower and upper ends, carrots were first cut into cylindrical segments approximately 30 mm in length. Each cylinder was then longitudinally divided into two equal halves. One half was used as the control (untreated), while the other half was subjected to plasma treatment. This approach allowed for a more accurate evaluation of the effect of plasma on nutritional properties, as each treated sample had a corresponding control derived from the same carrot section.

For microbial analyses, a single control group was used for both gas treatments, since all carrot surfaces had an equivalent area and geometry, minimizing variability in microbial load distribution. In contrast, for physicochemical quality analyses (β-carotene, ascorbic acid, TPC, TAA, color, and texture), separate control samples were prepared for each plasma gas type (argon and dry air), ensuring a precise comparison within treatment groups.

### 2.5. CAP Treatment

**Figure 1:**
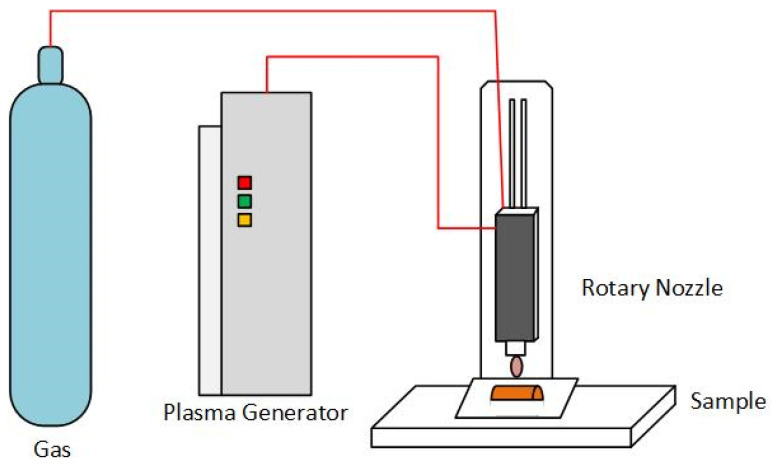
The open-air atmospheric pressure jet plasma system used in the study.

An open-air atmospheric pressure jet plasma system (Plasmatreat GmbH, Steinhagen, Germany) was used to treat the carrot surfaces. The plasma system consisted of a 1000 VA plasma generator (FG5001), a rotary plasma nozzle (RD2004), a high voltage transformer, and a pressure supply control unit (Figure 1).

#### 2.5.1. Evaluation of CAP Parameters

CAP parameters were evaluated for each process gas to select the conditions that provided the maximum decontamination level of *E. coli*-inoculated samples without exceeding the surface temperature of 50 °C to avoid possible heat-induced damage to the fresh-cut carrot surface. Dry air or high-purity argon (99.999%) with an inlet pressure of 300 kPa was used as the process gas for the plasma treatments. Three different jet nozzle to substrate distances were evaluated for each gas type: 10, 20 and 30 mm for argon and 20, 30 and 40 mm for dry air. Exposure times were set to 10, 30 and 60 s for both process gases. The surface temperature of the carrots was monitored using an infrared thermometer (VT 04, Fluke Corporation, Everett, WA, USA). The internal temperature of the carrot samples was also tracked with a probe thermometer (30.1048, TFA Dostmann, Germany). The most effective combination for each gas type was then selected and applied in subsequent physicochemical and microbiological evaluations.

#### 2.5.2. CAP Treatment of Fresh-Cut Carrots and Storage

Based on the obtained results, a 60 s exposure time was selected for both process gases. The jet nozzle to substrate distances of 10 mm and 30 mm were selected for argon and dry air, respectively. After plasma treatment, carrots were immediately vacuum packed in sterile polyethylene bags using a vacuum packaging machine (MV-20, Lipovak, Sakarya, Turkey) under 100 Pa and 2 s package sealing performed at 150 °C package closing conditions and stored for three weeks at 4 °C. Quality and microbiological analyses were performed on both of the control and plasma-treated halves of the carrot samples after 0, 1, 2 and 3 weeks of storage.

### 2.6. Microbial Load Examination

For the microbiological analyses, *E. coli*. ATCC 25922-inoculated carrot samples were used in order to assess the decontamination efficiency of the plasma treatments. To enumerate the *E. coli* count on carrot surfaces, carrots were placed in sterile stomacher bag with 100 mL phosphate-buffered saline (pH: 7.4) containing Tween 80 (1%, *v*/*v*) and agitated for 10 min. Then, 1 mL of the suspension from the appropriate dilutions was inoculated onto tryptone bile X-glucuronide agar using the pour plate method. The plates were incubated first for 4 h at 30 °C to enable recovery of injured bacteria and then for 20 h at 44 °C. The number of positive (blue-green) colonies on the plate was counted. Each sample group was analyzed using three independent fresh-cut carrot samples (*n* = 3).

### 2.7. Physicochemical Quality Analysis

For physicochemical quality analysis, non-inoculated fresh-cut carrot samples were used to avoid potential interference of microbial inoculum with the biochemical parameters being measured. Analyses of β-carotene, ascorbic acid, TPC, TAA, color and texture were performed comparing the non-plasma-treated half of each carrot slice (control sample) with the plasma-treated other half, separately for each week. Each treatment group was analyzed using three independent fresh-cut carrot samples (*n* = 3).

#### 2.7.1. β-Carotene Measurement

The β-carotene content of carrot samples was determined by an HPLC system (1200 Infinity HPLC, Agilent Technologies, Santa Clara, CA, USA) consisting of a quaternary pump (G1311C, Agilent), an autosampler (G1329B, Agilent), a column thermostat (G1316A, Agilent) and a DAD detector (G1315D Agilent). Analyses were carried out according to Ekesa, et al. [22] with slight modifications. The β-carotene in carrot samples was extracted with a 50 mL methanol/tetrahydrofuran (THF) (1:1, *v*/*v*) solution using a blender (8011, Waring, CT, USA) for 1 min. Then, the extract was filtered through a 0.45 µm nylon syringe filter. HPLC elution was carried out at 20 °C and utilized methanol/THF (95:5, *v*/*v*) at a flow rate of 0.8 mL min^−1^ as the mobile phase. The analytical column was Zorbax Eclipse plus C18 (5 µm, 250 mm × 4.6 mm) (Agilent Technologies, Santa Clara, CA, USA). The chromatogram was monitored simultaneously at 450 nm.

#### 2.7.2. Ascorbic Acid Measurement

The samples were homogenized with 1% phosphoric acid (H_3_PO_4_) in a proportion of 1:10 (*w*/*v*) using Ultra Turrax (T25, IKA, Staufen, Germany). The mixture was centrifuged at 11,068× *g* for 10 min at 4 °C. Then, the diluted sample was filtered through a 0.45 μm nylon syringe filter and immediately injected into the HPLC. A Zorbax PLRP-S column (5 μm, 250 mm × 4.6 mm) (Agilent Technologies, Santa Clara, CA, USA) was used for separation. HPLC elution was carried out at 35 °C and utilized deionized water (pH: 3, adjusted with H_3_PO_4_) at a flow rate of 0.5 mL min^−1^ as the mobile phase. The detection wavelength was 254 nm.

#### 2.7.3. Determination of TPC

TPC was measured according to Spanos and Wrolstad [23]. Approximately 4 g of sample was extracted with 25 mL of methanol and homogenized using an Ultra Turrax (T25, IKA, Staufen, Germany). The diluted solution was mixed with 5 mL Folin–Ciocalteu reagent and 4 mL saturated Na_2_CO_3_ solution, then held in a water bath at 45 °C for 5 min. The absorbance of the solution was measured at 760 nm using a spectrophotometer (UV-1800, Shimazdu, Tokyo, Japan). Results were expressed as gallic acid (mg 100 g^−1^).

#### 2.7.4. Determination of TAA

TAA was determined by Trolox Equivalent Antioxidant Capacity (TEAC) method according to Re, et al. [24]. ABTS^+^ radical cation was generated by reacting 7 mM ABTS with 2.45 mM potassium persulfate in the dark at room temperature (22 ± 1 °C) for 16 h. Three mL of ABTS^+^ cation solution was mixed with 30 μL methanolic extract (4 g sample-25 mL methanol). After 6 min. of incubation, the decrease in absorbance was recorded at 734 nm. The TAA was expressed as TEAC (mmol Trolox 100 g^−1^ carrot).

#### 2.7.5. Color Measurement

Color measurements were performed using the L* a* b* color space (CIELab space) with a CM-5 Spectrophotometer (Konica Minolta Sensing, Inc., Osaka, Japan). The illuminant used was D65 and the observer angle was 10°. L* (lightness or brightness; 0 = black, 100 = white), a* (−a*; greenness, +a*; redness) and b* (−b*; blueness, +b*; yellowness) measurements were performed in triplicate and each color reading was recorded on three equidistant points of each replicate, to record the average value. The whiteness index (WI) was calculated according to Equation (1) [25]:

(1) $$WI=100-\sqrt{\left[{\left(100-L\right)}^{2}+a^{2}+b^{2}\right]}$$

Total color differences (Δ*E*) were calculated according to Equation (2) by using the non-plasma-treated half of each carrot slice as a reference individually:

(2) $$∆E=\sqrt{\left[{\left(L-L_{0}\right)}^{2}+{\left(a-a_{0}\right)}^{2}+{\left(b-b_{0}\right)}^{2}\right]}$$

#### 2.7.6. Texture Analysis

Texture analysis of carrot samples was performed using a texture analyzer (TA.HD plus, Stable Micro Systems, Godalming, UK) with a 5 kg load cell. The firmness of the carrots was measured via a penetration test using a 2 mm diameter stainless steel flat cylinder probe (*p*/2). The approach and test speed of the probe were 1 mm s^−1^ and the penetration distance was 5 mm. For each experiment, the maximum penetration force (N) was recorded as an indicator of carrot firmness. Analyses were performed in triplicate and each sample was measured five times by the instrument.

### 2.8. Statistical Analysis

Each assay was performed using three independent carrot samples (*n* = 3). For texture analysis, three independent carrot samples were used per treatment group, and measurements were taken from five different points on each sample. The results are given as mean ± standard deviation (SD). One-way analysis of variance (ANOVA) was used to compare the means. Post hoc multiple comparisons were determined by Duncan’s multiple range test with the level of significance set at *p* < 0.05. All statistical analyses were performed using SPSS 17.0 (SPSS Inc., Chicago, IL, USA).

## 3. Results and Discussion

### 3.1. Evaluation of CAP Process Parameters for Microbial Inactivation

To evaluate the effects of argon and dry air CAP treatments on the decontamination efficacy of fresh-cut carrot samples comparatively, *E. coli* was inoculated onto carrot surfaces with an initial concentration of 10^7^ CFU g^−1^, and cell viability immediately after treatment was measured. In general, the effectiveness of the argon CAP treatment was increased significantly (*p* < 0.05) with extended exposure time, regardless of the jet nozzle to substrate distance used (Table 1). On the other hand, shorter distances resulted in higher reductions in the population of *E. coli* at the same plasma duration period, which is possibly related to the increased concentration of photons and reactive species that can reach the surface [26]. A reduction of 4.54 log CFU g^−1^, which is satisfactory from the point of view of decontamination, was achieved after 60 s of plasma treatment applied at a 10 mm of distance. Butscher, et al. [27] reported an inactivation between 1.4 and 3.4 log on *E. coli* counts of inoculated alfalfa, onion, radish and cress seeds after 10 min of argon CAP treatment.

A similar trend was observed for dry-air plasma treatment. In general, higher reduction efficiencies were obtained with extended exposure time and decreased jet nozzle to substrate distances (Table 2). The surface temperature exceeded 50 °C either at prolonged plasma treatment at a constant distance or shorter distances from the nozzle at the same plasma duration. For example, a 5.05 log reduction in *E. coli* count was achieved after a 60 s treatment at a 20 mm distance, and the surface temperature of the carrots reached 73.1 °C, which is above the threshold for thermal inactivation of *E. coli* [26]. For this reason, the parameters (30 mm, 60 s) enabling the second best reduction (4.20 log) were selected for further studies. The change in central temperature of the carrots was not significant (*p* > 0.05).

In comparison with argon CAP, dry air plasma was more efficient under the same process conditions, enabling a 1.4–3.2 log higher reduction in *E. coli* count. Similar observations have been reported in studies suggesting that the type of working gas plays a critical role in plasma efficacy. Than, et al. [28] showed that air plasma had better bactericidal effectiveness than argon CAP against *Diutina catenulate* isolated from longan fruit. The higher efficiency of air plasma was also shown in inactivating *Salmonella enteritidis* PT30 on almonds compared with plasma generated with N_2_, O_2_, CO_2_ and CO_2_ in an admixture with argon [29].

According to Deng, et al. [30], inactivation of microorganisms via plasma can be due to the effect of heat, charged particles, electric fields, UV photons and some reactive species such as atomic oxygen, metastable oxygen molecules, ozone and hydroxyl radicals, which are commonly found in a gas discharge. Air plasmas are excellent sources of reactive oxygen species (ROS) and reactive nitrogen species (RNS) such as atomic oxygen, O_3_, OH radicals, NO, NO_2_^−^, etc., which have a direct impact on the cells of microorganisms, particularly on their outermost membranes [31]. In the case of atmospheric argon CAP, these reactive species can only be generated upon contact with air [32], which limits their effectiveness as compared with air plasma.

Based on the obtained results, for argon, a jet nozzle to substrate distance of 10 mm and 60 s exposure time and for dry air, a distance of 30 mm and 60 s exposure time was selected for further quality evaluation studies. The fresh-cut carrot samples were plasma-treated with argon and dry air separately using previously determined conditions, and changes in microbiological and nutritional characteristics were comparatively investigated over a three-week cold storage period.

### 3.2. Microbiological and Physicochemical Assessment of Plasma-Treated Carrots During Storage

#### 3.2.1. Microbiological Quality During Storage

No changes were noticed in the *E. coli* count of the untreated fresh-cut carrot samples after 1 week of storage (Table 3). However, a continuous decrease in cell number was observed over the extended storage period and a slight but statistically significant reduction of 8% was recorded after 3 weeks. A higher reduction rate was observed in argon CAP treated fresh-cut carrot samples throughout storage and significantly fewer *E. coli* cells were detected even after 1 week of storage. By further increasing the storage period to 2 and 3 weeks, reductions of 37% and 53% were achieved, respectively.

The decrease in *E. coli* count of cold atmospheric dry air plasma-treated samples was even higher during storage. Complete inhibition of *E. coli* cells was recorded after 1 week of storage. The phenomenon of progressive inhibition of the microorganisms during storage can be attributed to the continued action of plasma-generated reactive species. The storage of plasma-treated samples facilitates the diffusion of the generated reactive species into the surrounding fluids [33].

Surowsky, et al. [34] reported only a 1 log reduction in *Citrobacter freundii* load in apple juice following immediate plating after 480 s of plasma exposure, while the reduction increased to a level of 4.4 log and 5.1 log cycles after 3 h and 24 h storage. In our study, *E. coli* counts on plasma-treated samples did not decrease sharply during the storage period, as observed in the apple juice samples. This may be due to the limited diffusion of reactive species in a solid matrix such as carrots. CAP exhibits differential antimicrobial efficacy between liquid and solid foods, In liquids, reactive species can be in full contact with the liquid medium, whereas in solid foods, treatment efficiency is restricted by surface characteristics, porosity, and moisture content, which restrict the penetration depth of the reactive species [35].

#### 3.2.2. Physicochemical Quality During Storage

##### Surface Color Change

The white discoloration of fresh-cut carrots’ surfaces during refrigerated storage is considered as an index representing the freshness of the products. The higher WI value indicates the more severe white discoloration [36]. The white discoloration on minimally processed carrots is correlated with both surface dehydration (inducing a reversible color change) and lignin formation (resulting in an irreversible color change), as responses to wounding (peeling, cutting, etc.). No significant changes were found in the WI value of fresh-cut carrot surfaces following argon CAP treatment (Table 4), indicating that there was no significant loss of moisture that could cause white discoloration of carrots. CAP can influence the color profile of food products, potentially affecting consumer acceptance. However, studies have shown that CAP generally maintains the instrumental color parameters (L*, a*, and b*) of foods, indicating minimal impact on the visual appearance of β-carotene-rich products [17,37].

During the storage period, the differences between the WI values of argon plasma-treated and untreated samples were not statistically significant (*p* > 0.05). The WI of both the argon-treated and untreated samples slightly increased throughout the refrigerated storage period and became noticeable only after 2 weeks. In the case of dry air CAP, a significant increase (*p* < 0.05) in the WI (3.7%) was observed in fresh-cut carrot surfaces, showing a minor degree of surface dehydration. Wang, Nian, Wu, Feng, Zhang, Zhang, Zhu, Becker and Fang [17] also reported a significant loss of color in cucumber and carrot slices following CAP treatment. In another study, fresh-cut carrot discs were sealed in plastic packages treated by high-voltage DBD and stored for 24 h (8 °C with 90% relative humidity). An increase in L*, together with decreases in a* and b* values, considered as whitening indicators, was reported, where the a* and b* values of the treated plasma samples were lower than those of the control.

Similar to argon plasma-treated samples, a slightly increasing trend was recorded during storage, reaching only about 6% by week 3. Using a microwave-driven air plasma torch within a remote exposure chamber for indirect treatment for up to 10 min, Baier, et al. [38] reported a delayed strong alteration in color parameters of whole pieces of carrots after 24 h and 48 h storage, characterized by visible superficial browning.

**Figure 2:**
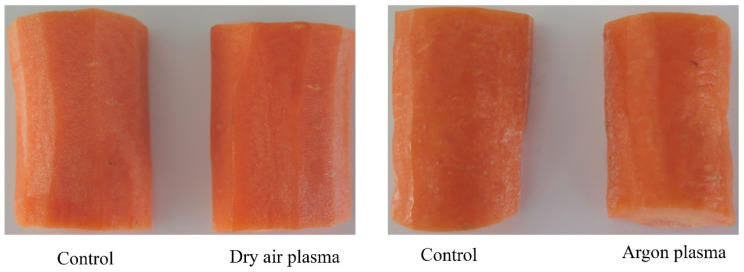
Example images of control and plasma-treated fresh-cut carrots acquired after 2 weeks of storage (4 °C).

According to the small Δ*E* between the untreated and the plasma-treated samples, it can be concluded that there were no visual apparent differences (Figure 2) in the color of fresh-cut samples, since Δ*E* values of 1.5–3.0 indicate barely noticeable color difference [39].

##### β-Carotene Content

**Figure 3:**
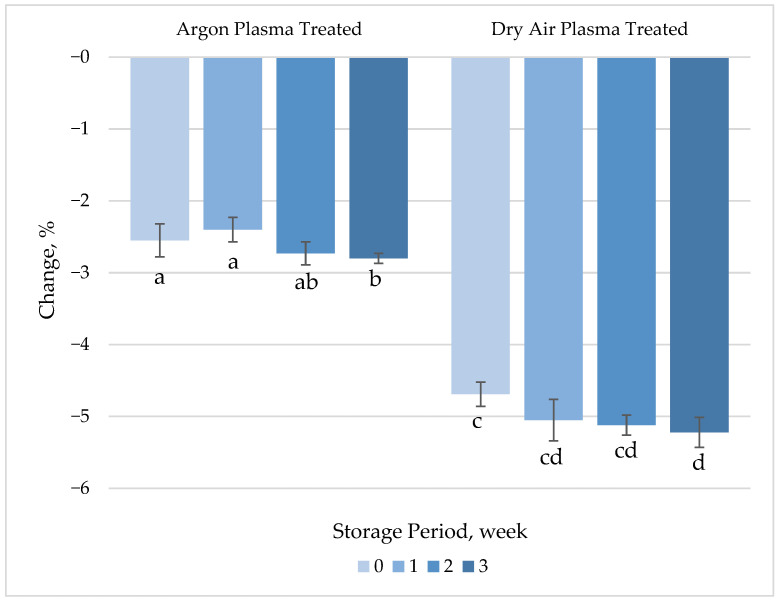
Percentage change in β-carotene concentration of plasma-treated fresh-cut carrots relative to their respective untreated controls during 3 weeks of refrigerated storage at 4 °C. Vertical bars represent the standard deviations of the means (*n* = 3). Different letters above the bars indicate statistically significant differences at *p* < 0.05.

In general, carotenoids have been reported to be stable under processing and storage conditions. It has previously been shown that the β-carotene levels of carrots are not affected by blanching at 60 °C for 40 min [40]. However, the characteristic conjugated polyene chain of β-carotene makes it susceptible to oxidation, which is highly influenced by the initiating agent. Carotenoids are abundantly present in the phloem tissue of carrots but become susceptible to degradation by air and light following removal of the epidermis during peeling. CAP treatment with dry air caused a slight (4.7%) but statistically significant (*p* < 0.05) reduction in the β-carotene content of fresh-cut carrot samples (Figure 3, Table S1), most likely mediated by plasma-generated oxidizing species, as the surface temperature did not exceed 50 °C during the treatment. A 2.6% decrease, which was not statistically significant (*p* > 0.05), was recorded in the β-carotene levels of fresh-cut carrots following argon plasma treatment.

In this study, a very slight decrease in β-carotene was achieved due to the limited penetration depth of the plasma components. Similar observations have been reported in other plasma-treated produce. Yi, Wang, Xiang, Yun, Pan, Jiang and Zhang [37] observed degradation of β-carotene and other antioxidant compounds in fresh-cut mango after CAP treatment, which was attributed to oxidative degradation caused by ROS and RNS. The loss of β-carotene appears to be limited by the plasma exposure conditions. Ramazzina, et al. [41] showed that up to 20 min of CAP treatment had no significant effect on the carotenoid content of kiwi samples compared to control samples. In contrast, Santos Jr, et al. [42] reported up to a 50% loss in total carotenoids of pumpkin puree treated with CAP corona discharge under continuous agitation using Ar as the process gas, depending on the plasma duration. These findings indicate that the impact of CAP on carotenoids is highly dependent on the specific plasma parameters applied (e.g., gas composition, exposure time, and energy input), as well as the physicochemical characteristics of the treated substrate, including its tissue structure, water content, and surface composition.

The interaction of CAP with food matrices can influence the stability and bioavailability of bioactive compounds, including β-carotene. Carotenoid levels in foods may either increase or decrease following plasma treatment [43]. In some food products, prolonged CAP treatment has been associated with a reduction in beta-carotene content due to excessive exposure to reactive species and oxidative reactions [44]. Conversely, it has been reported that plasma treatment can increase the free carotenoid content by breaking the bond between carotenoid molecules and cell membranes via reactive species [45].

##### Ascorbic Acid Content

**Figure 4:**
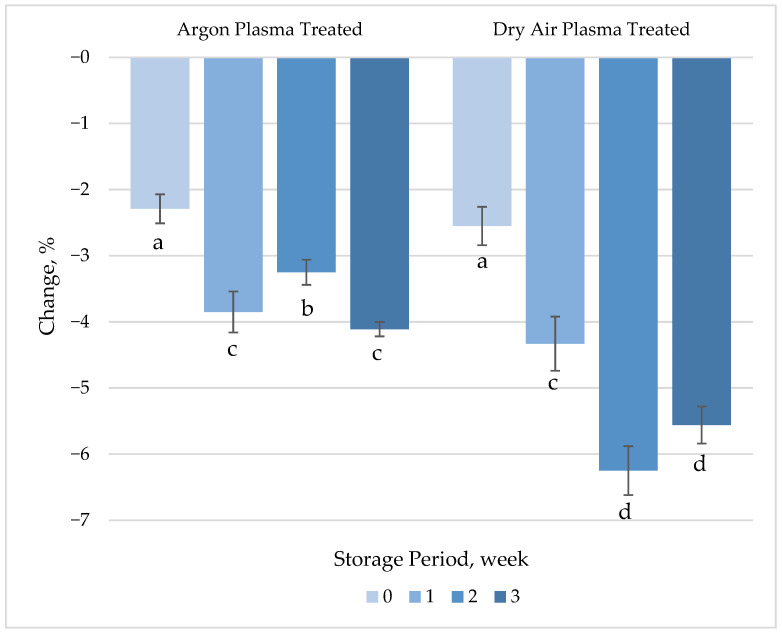
Percentage change in ascorbic acid concentration of plasma-treated fresh-cut carrots relative to their respective untreated controls during 3 weeks of refrigerated storage at 4 °C. Vertical bars represent the standard deviations of the means (*n* = 3). Different letters above the bars indicate statistically significant differences at *p* < 0.05.

The ascorbic acid loss with the immediate effect of both the argon (2.3%) and dry air (2.6%) plasma treatment was not statistically significant (Figure 4, Table S1). These insignificant changes may be attributed to the short treatment duration and limited energy input used in this study, but more likely to the surface-restricted effect of plasma on the product. In agreement with our findings, Wang, Nian, Wu, Feng, Zhang, Zhang, Zhu, Becker and Fang [17] reported only a slight reduction (3.2%) in the vitamin C content of carrot slices after atmospheric plasma treatment. However, an earlier study by Bozkurt, et al. [46] reported a 30% loss in vitamin C in water solutions with the effect of 1 min dielectric barrier discharge plasma treatment using a He-O_2_ gas mixture. Since plasma was applied only to the surface of the product in this study, any chemical reaction that is responsible for the loss in ascorbic acid content would primarily occur at the surface level [47].

Oxidation by reactive oxygen species, particularly ozone, has been proposed as the main mechanism underlying ascorbic acid loss during plasma treatment [48]. It has been hypothesized that ascorbic acid degrades either by direct attack of ozone, described by the Criegee mechanism (producing ozonides), or through indirect oxidation via secondary species such as singlet oxygen and excited molecular oxygen [48]. Additionally, UV radiation generated by plasma may also play an important role in ascorbic acid degradation [17]. The significant increase in the difference between ascorbic acid levels in untreated and plasma-treated samples during refrigerated storage was associated with progressive oxidative degradation. Peeling and removal of the periderm layer may lead to the leakage of tissue fluids, exposing internal tissues and making disrupted cell walls more susceptible to oxidation and enzymatic changes [49]. Similarly, a continuous decrease in ascorbic acid content caused by peeling has been reported during refrigerated storage of fresh-cut carrots [50]. The difference among the process gases used became significant (*p* < 0.05) after 2 weeks of storage, with ascorbic acid loss being approximately 5–6% greater in treated samples compared to controls.

##### TPC

**Figure 5:**
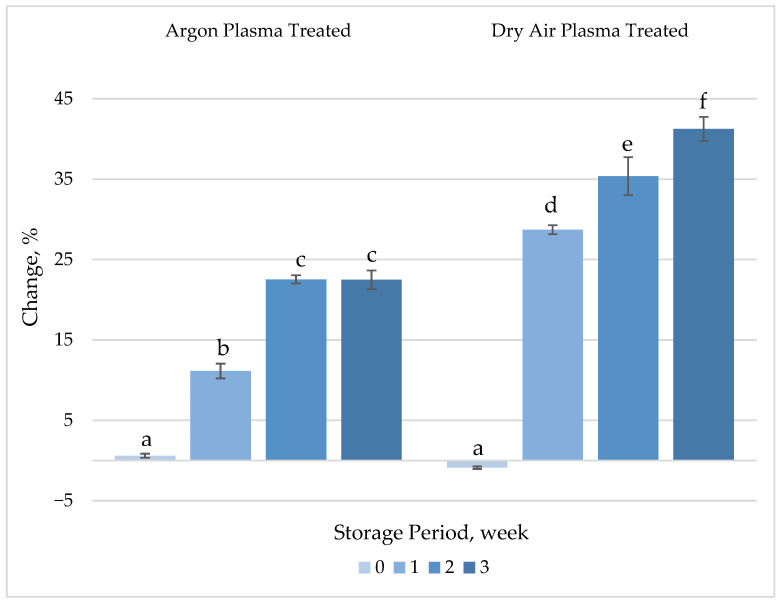
Percentage change in total phenolic content (TPC) of plasma-treated fresh-cut carrots relative to their respective untreated controls during 3 weeks of refrigerated storage at 4 °C. Vertical bars represent the standard deviations of the means (*n* = 3). Different letters above the bars indicate statistically significant difference at *p* < 0.05.

Neither of the plasma treatments resulted in statistically significant changes (*p* > 0.05) in the TPC of fresh-cut carrots (Figure 5, Table S1). However, the TPC increased significantly (*p* < 0.05) compared to control samples throughout the storage period. The increase was more pronounced in air plasma-treated samples, reaching up to a 41% higher than untreated samples after 3 weeks of storage. CAP treatment was also shown to significantly influence the TPC of maize kernels [51]. As working pressure and treatment time increased, a progressive enhancement in TPC was observed. Specifically, TPC reached 2.63 mg g^−1^ in Ar plasma-treated samples and 2.79 mg g^−1^ in N_2_ plasma-treated samples, compared to 2.14 mg g^−1^ and 2.16 mg g^−1^ in their respective untreated controls (*p* < 0.05). This enhancement may be attributed to the plasma-induced deactivation of oxidative enzymes such as polyphenol oxidase (PPO) and peroxidase (POD), both of which utilize phenolic compounds as substrates.

ROS are generated in normal metabolic processes as by-products of cell metabolism and are actively involved in activation of phenylalanine ammonia lyse and accumulation of phenolic compounds [52]. ROS generated during the plasma treatment may induce biosynthesis and accumulation of phenolics, resulting in noticeable alterations in TPC throughout the storage period as responses to stress factors. Dry air plasma exhibited a more pronounced effect on TPC, which may be attributed to the higher concentration of plasma-generated ROS, as previously described.

##### TAA

**Figure 6:**
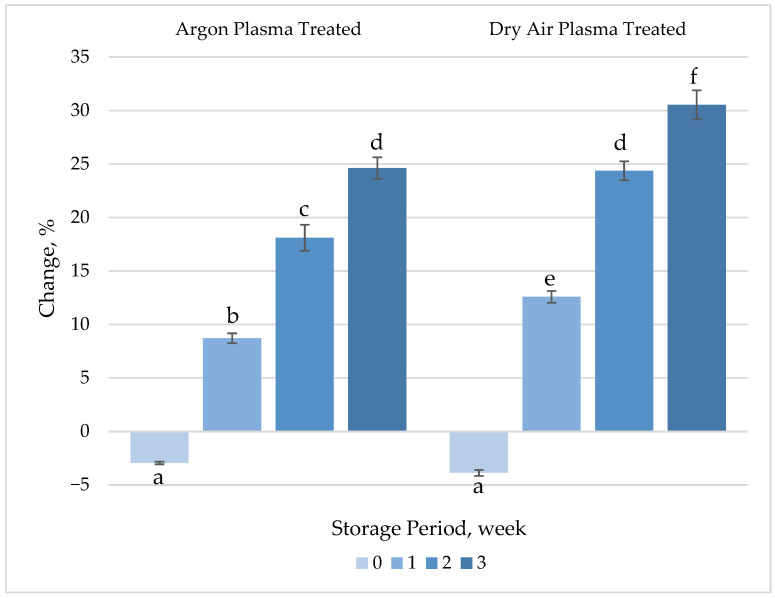
Percentage change in total antioxidant activity (TAA) of plasma-treated fresh-cut carrots relative to their respective untreated controls during 3 weeks of refrigerated storage at 4 °C. Vertical bars represent the standard deviations of the means (*n* = 3). Different letters above the bars indicate statistically significant differences at *p* < 0.05.

No significant changes (*p* > 0.05) were observed in the TAA of fresh-cut carrots after plasma treatment (Figure 6, Table S1). These findings are consistent with the results obtained for ascorbic acid, β-carotene and TPC, the main compounds contributing antioxidant activity of carrots. However, a gradual increase in the activity was observed between the untreated and treated samples after 1 week of refrigerated storage. The significant increase (*p* < 0.05) in TAA despite the lack of change or slight decreases observed in ascorbic acid and β-carotene contents during the storage period may be attributed to the increase in TPC. Indeed, the alteration in TAA showed a similar trend as TPC throughout the 3 weeks of refrigerated storage.

In agreement with our findings, Heredia and Cisneros-Zevallos [53] correlated the increase in the antioxidant capacity of carrots after wounding with an increase in TPC. Li, et al. [54] reported that CAP treatment could effectively enhance the antioxidant activity of fresh-cut pitaya fruit. In their study, the fruit samples were treated with dielectric barrier discharge (DBD) plasma at 60 kV for 5 min and then stored for 36 h. The antioxidant activity of the plasma-treated samples increased by 87.71%, compared to 78.13% in the untreated control group. In a recent study, CAP treatment using argon and nitrogen gases was found to significantly enhance the antioxidant capacity of maize kernels. The DPPH scavenging activity increased 1.31-fold for Argon plasma and 1.25-fold for N_2_ plasma compared to untreated controls. Throughout the storage period, CAP-treated samples maintained significantly higher DPPH scavenging activity than the untreated samples (*p* < 0.05), with Argon plasma-treated maize consistently exhibiting superior antioxidant activity compared to nitrogen plasma-treated samples [51].

##### Texture

**Figure 7:**
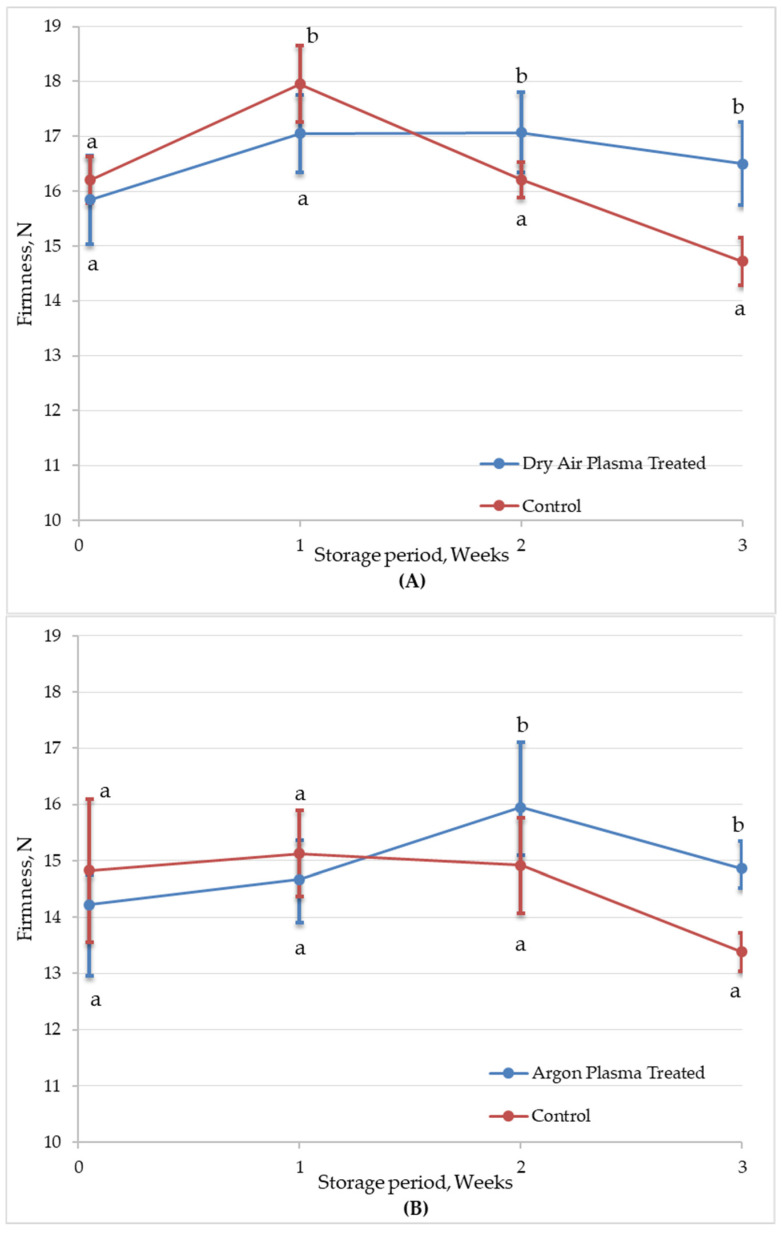
Firmness (N) of untreated (control) and plasma-treated ((**A**): Dry air; (**B**): Argon) fresh-cut carrot samples during 3 weeks of refrigerated (4 °C) storage. Vertical bars represent the standard deviations of the means (*n* = 15). Different letters above each sampling point indicate statistically significant differences at *p* < 0.05.

Texture is one of the most important sensorial quality attributes for consumer acceptance of fresh-cut carrots. It influences not only the overall eating experience but also plays a significant role in perceived freshness and quality, thereby impacting consumer preferences. The reduction in firmness during the storage of fresh-cut produce is typically associated with textural degradation and is widely recognized as a key indicator of declining product quality [55]. Figure 7 shows changes in the firmness of untreated and plasma-treated fresh-cut carrot samples during 3 weeks of refrigerated storage. Atmospheric CAP treatment did not affect the texture of fresh-cut carrots in terms of firmness, regardless of the type of process gas used. The firmness values of both the plasma-treated samples slightly increased during the first week of storage, which may be attributed to mild surface dehydration resulting in increased tissue rigidity. After the first week, a clear firmness reduction was observed in untreated samples, possibly due to increased enzymatic and microbial activity. Firmness continued to decline, reaching approximately 9% loss by the end of the storage period. In contrast, plasma-treated samples effectively maintained the texture of the fresh-cut carrots throughout storage. The better preservation of texture with plasma effect can be attributed to reduced microbial deterioration during storage.

In contrast to control samples, plasma-treated samples maintained their firmness throughout the storage period. This enhanced texture retention can be attributed to both reduced microbial activity and potential cross-linking of cell wall polymers induced by plasma-generated reactive species [56]. Additionally, plasma treatment can inactivate or reduce the activity of enzymes such as PPO and POD, which are responsible for enzymatic browning and softening of fruits and vegetables [57,58].

The duration and intensity of plasma treatment, along with the physical and structural attributes of the substrate, are key determinants of texture and firmness changes in plasma-treated foods. Similarly to our findings, Wu, et al. [59] reported that CAP treated cherries could retain their firmness during six days of storage. However, with the longer plasma treatment, the firmness of the cherry samples was significantly reduced because of tissue damage. In another study, CAP-treated fresh-cut Hami melons showed no significant change in firmness during storage [56]. Zhang, et al. [60] reported that the effect of CAP on the texture of fresh-cut pears was closely linked to the plasma treatment parameters. Notably, a treatment at 65 kV for 5 min resulted in a significant firmness loss after just 3 days of storage, which continued to decline over time. This deterioration was attributed to superficial tissue damage and internal structural breakdown caused by high plasma intensity. In contrast, lower intensity treatments (45 kV for 1 min) did not negatively affect firmness, and in some cases, samples treated with these mild conditions displayed higher firmness values than the control after five days of storage. These findings suggest that increased plasma power and exposure time can exacerbate softening, whereas moderate plasma parameters may help preserve or even enhance firmness, likely through microbial load reduction and reduced enzymatic degradation.

## 4. Conclusions

CAP treatment represents a promising non-thermal technology for improving the safety and extending the shelf-life of fresh-cut produce. This study demonstrated that a 60 s treatment with either argon or dry air plasma effectively inactivated *E. coli* on fresh-cut carrot surfaces (>4 log CFU g^−1^ reduction) while preserving essential quality characteristics. In general, dry air plasma was more efficient than argon plasma under the same process conditions.

Beyond microbial decontamination, plasma treatment provided significant benefits during storage, including continued microbial inhibition, prevention of quality deterioration, and enhancement of bioactive compounds. The observed increases in TPC and TAA in plasma-treated samples indicate that controlled stress responses can be leveraged to enhance nutritional quality.

From a practical perspective, the CAP technology investigated in this study offers several advantages for the fresh-cut produce industry: it requires no chemical inputs, leaves no residues, operates at ambient conditions, and has minimal impact on product quality. The short treatment times (60 s) make it compatible with existing processing lines, and the use of air as a process gas reduces operational costs compared to noble gas systems.

The overall results of this study showed that CAP treatment, especially dry air plasma, can be considered a versatile tool with higher economic feasibility for rapid non-thermal surface decontamination of fresh-cut vegetables, enabling the advantage of maintaining the original characteristics of fresh produce. Further research should focus on scaling this technology for industrial implementation, optimizing treatment parameters for different produce types, and investigating consumer acceptance of plasma-treated products. The combination of plasma treatment with complementary preservation technologies within a hurdle concept may provide additional synergistic benefits for extending the shelf-life of minimally processed fruits and vegetables.

## Figures and Tables

**Table 1:** Effects of argon CAP treatment parameters on *E. coli* ATCC 25922 counts and temperatures in fresh-cut carrots.

Gas	Jet Nozzle to Substrate Distance (mm)	Exposure Time (s)	Microbial Reduction (log CFU g^−1^)	Surface Temperature (°C)	Central Temperature (°C)
Argon	10	10	1.73 ± 1.41 B	26.1 ± 1.0 C	19.1 ± 0.5 A
		30	1.93 ± 0.14 B	32.4 ± 0.9 E	19.5 ± 0.7 A
		60	4.54 ± 0.14 A	42.3 ± 0.3 F	20.0 ± 0.9 A
	20	10	0.79 ± 0.03 B	22.7 ± 1.0 B	19.0 ± 0.3 A
		30	0.73 ± 0.01 B	28.0 ± 1.4 D	19.6 ± 0.4 A
		60	1.84 ± 0.09 B	32.2 ± 1.7 E	20.1 ± 0.6 A
	30	10	0.44 ± 0.02 B	20.6 ± 0.6 A	18.7 ± 0.4 A
		30	0.60 ± 0.11 B	24.9 ± 0.4 C	19.0 ± 0.5 A
		60	1.04 ± 0.13 B	28.2 ± 0.4 D	19.6 ± 0.8 A

Surface temperature and central temperature of carrots before plasma treatment: 20.0 ± 0.5 °C and 19.2 ± 0.4 °C, respectively. Data are mean values ± standard deviation (*n* = 3). Values followed by different letters in the same column are significantly different (*p* < 0.05).

**Table 2:** Effects of dry air CAP treatment parameters on *E. coli* ATCC 25922 counts and temperatures in fresh-cut carrots.

Gas	Jet Nozzle to Substrate Distance (mm)	Exposure Time (s)	Microbial Reduction (log CFU g^−1^)	Surface Temperature (°C)	Central Temperature (°C)
Dry Air	20	10	2.29 ± 0.23 BC	46.0 ± 2.8 C	20.3 ± 2.2 A
		30	3.43 ± 0.19 E	59.1 ± 1.5 D	20.6 ± 1.7 A
		60	5.05 ± 0.11 F	73.1 ± 1.6 E	23.4 ± 1.5 A
	30	10	1.85 ± 0.27 B	36.6 ± 3.0 B	19.5 ± 0.4 A
		30	3.07 ± 0.47 CDE	46.0 ± 2.1 C	19.9 ± 0.3 A
		60	4.20 ± 0.33 EF	49.4 ± 2.5 C	22.1 ± 1.3 A
	40	10	0.89 ± 0.16 A	32.0 ± 2.3 A	20.5 ± 0.4 A
		30	1.34 ± 0.16 AB	36.5 ± 0.7 B	21.2 ± 1.2 A
		60	2.35 ± 0.29 BCD	44.0 ± 1.4 C	20.8 ± 0.4 A

Surface temperature and central temperature of carrots before plasma treatment: 20.0 ± 0.5 °C and 19.2 ± 0.4 °C, respectively. Data are mean values ± standard deviation (*n* = 3). Values followed by different letters within the same column are significantly different (*p* < 0.05).

**Table 3:** Survival of *E. coli* ATCC 25922 on control and plasma-treated fresh-cut carrots * during 3 weeks of refrigerated (4 °C) storage.

Sample	*E. coli* ATCC 25922 Counts (log CFU g^−1^) During the Storage			
	0th Week	1st Week	2nd Week	3rd Week
Control (inoculated)	6.92 ± 0.11 Aa	6.96 ± 0.15 Aa	6.42 ± 0.24 Ab	6.03 ± 0.11 Ac
Argon Plasma (10 mm-60 s)	2.65 ±0.12 Ba	1.92 ± 0.15 Bb	1.67 ± 0.19 Bbc	1.26 ± 0.20 Bd
Dry Air Plasma (30 mm-60 s)	2.34 ± 0.21 Ba	<1 *	<1	<1

* No *E. coli* was detected (<1 log CFUg^−1^) on fresh uninoculated carrots. Data are mean values ± standard deviation (*n* = 3). Values followed by different capital letters within the same column are significantly different (*p* < 0.05). Values followed by different lowercase letters within the same raw are significantly different (*p* < 0.05).

**Table 4:** Variation in total color difference (Δ*E*) and whiteness index (WI) values of control and plasma-treated fresh-cut carrots during the refrigerated (4 °C) storage.

Storage (Week)	Argon Plasma			Dry Air Plasma		
	Δ*E*	WI		Δ*E*	WI	
		Control	Plasma-Treated		Control	Plasma-Treated
0	2.88 ± 0.80 A	36.38 ± 0.65 Aa	36.74 ± 0.48 Aa	3.03 ± 0.56 A	36.51 ± 0.53 Aa	38.47 ± 0.35 Ab
1	1.63 ± 0.57 A	36.71 ± 0.34 Aa	37.22 ± 0.31 Aa	2.19 ± 0.41 A	36.29 ± 0.65 Aa	38.94 ± 0.54 Bb
2	2.05 ± 0.23 A	37.81 ± 0.22 Ba	38.13 ± 0.29 Ba	2.03 ± 0.64 A	37.36 ± 0.18 Ba	39.55 ± 0.37 Bb
3	1.85 ± 0.41 A	38.45 ± 0.37 Ca	38.88 ± 0.14 Ca	2.14 ± 0.96 A	38.83 ± 0.26 Ca	40.74 ± 0.21 Cb

Data are mean values ± standard deviation (*n* = 3). Values followed by different capital letters within the same column are significantly different (*p* < 0.05). Values followed by different lowercase letters within the same raw between control and treated samples of the same treatment are significantly different (*p* < 0.05).

## Data Availability

The original contributions presented in the study are included in the article, further inquiries can be directed to the corresponding author.

## Supplementary Materials

The following supporting information can be downloaded at: https://www.mdpi.com/article/10.3390/foods14091599/s1, The supplementary file contains the data of quality analysis of carrot samples.

## Abbreviations

The following abbreviations are used in this manuscript:

CAP	Cold atmospheric plasma
TPC	Total phenolic content
TAA	Total antioxidant activity
ROS	Reactive oxygen species
ROS	Reactive nitrogen species
PPO	Polyphenol oxidase
POD	Peroxidase

## References

[1] Ramos B., Miller F., Brandão T.R., Teixeira P., Silva C.L. (2013). Fresh fruits and vegetables—An overview on applied methodologies to improve its quality and safety. Innov. Food Sci. Emerg. Technol..

[2] Ying D., Sanguansri L., Cheng L., Augustin M.A. (2021). Nutrient-dense shelf-stable vegetable powders and extruded snacks made from carrots and broccoli. Foods.

[3] Dallagi W., Rguez S., Hammami M., Bettaieb Rebey I., Bourgou S., Hamrouni Sellami I. (2023). Optimization of processing conditions to enhance antioxidant and carotenoid contents of carrot juice. J. Food Meas. Charact..

[4] Ahmad T., Cawood M., Iqbal Q., Ariño A., Batool A., Tariq R.M.S., Azam M., Akhtar S. (2019). Phytochemicals in *Daucus carota* and their health benefits. Foods.

[5] Tappi S., Gozzi G., Vannini L., Berardinelli A., Romani S., Ragni L., Rocculi P. (2016). Cold plasma treatment for fresh-cut melon stabilization. Innov. Food Sci. Emerg. Technol..

[6] Raja P., Abitha M., Ganapathy S. (2022). Enhancing shelf-life of carrot (*Daucus carota*) during post-harvest processing. Curr. Hortic..

[7] Becaro A.A., Puti F.C., Panosso A.R., Gern J.C., Brandão H.M., Correa D.S., Ferreira M.D. (2016). Postharvest quality of fresh-cut carrots packaged in plastic films containing silver nanoparticles. Food Bioprocess Technol..

[8] Santos M.I.S., Marques C., Mota J., Pedroso L., Lima A. (2022). Applications of essential oils as antibacterial agents in minimally processed fruits and vegetables—A review. Microorganisms.

[9] Kangas S., Takkinen J., Hakkinen M., Nakari U.-M., Johansson T., Henttonen H., Virtaluoto L., Siitonen A., Ollgren J., Kuusi M. (2008). *Yersinia pseudotuberculosis* O:1 traced to raw carrots, Finland. Emerg. Infect. Dis..

[10] Sivapalasingam S., Friedman C.R., Cohen L., Tauxe R.V. (2004). Fresh produce: A growing cause of outbreaks of foodborne illness in the United States, 1973 through 1997. J. Food Prot..

[11] Orsat V., Gariepy Y., Raghavan G., Lyew D. (2001). Radio-frequency treatment for ready-to-eat fresh carrots. Food Res. Int..

[12] Cho J.-L., Kim C.-K., Park J., Kim J. (2017). Efficacy of aerosolized chlorine dioxide in reducing pathogenic bacteria on washed carrots. Food Sci. Biotechnol..

[13] Reineke K., Langer K., Hertwig C., Ehlbeck J., Schlüter O. (2015). The impact of different process gas compositions on the inactivation effect of an atmospheric pressure plasma jet on *Bacillus spores*. Innov. Food Sci. Emerg. Technol..

[14] Ramkumar R., Arun Prasath V., Karpoora Sundara Pandian N., Patra A., Sharma P., Arulkumar M., Sivaranjani S., Govindarasu P. (2024). Investigating the influence of pin-to-plate atmospheric cold plasma on the physiochemical, nutritional, and shelf-life study of two raisins varieties during storage. J. Food Meas. Charact..

[15] Moisan M., Barbeau J., Crevier M.-C., Pelletier J., Philip N., Saoudi B. (2002). Plasma sterilization. Methods and mechanisms. Pure Appl. Chem..

[16] Gök V., Aktop S., Özkan M., Tomar O. (2019). The effects of atmospheric cold plasma on inactivation of *Listeria monocytogenes* and *Staphylococcus aureus* and some quality characteristics of pastırma—A dry-cured beef product. Innov. Food Sci. Emerg. Technol..

[17] Wang R., Nian W., Wu H., Feng H., Zhang K., Zhang J., Zhu W., Becker K., Fang J. (2012). Atmospheric-pressure cold plasma treatment of contaminated fresh fruit and vegetable slices: Inactivation and physiochemical properties evaluation. Eur. Phys. J. D.

[18] Birania S., Attkan A.K., Kumar S., Kumar N., Singh V.K. (2022). Cold plasma in food processing and preservation: A review. J. Food Process Eng..

[19] Feizollahi E., Misra N., Roopesh M. (2021). Factors influencing the antimicrobial efficacy of dielectric barrier discharge (DBD) atmospheric cold plasma (ACP) in food processing applications. Crit. Rev. Food Sci. Nutr..

[20] Mahnot N.K., Siyu L.-P., Wan Z., Keener K.M., Misra N. (2020). In-package cold plasma decontamination of fresh-cut carrots: Microbial and quality aspects. J. Phys. D Appl. Phys..

[21] Silvetti T., Pedroni M., Brasca M., Vassallo E., Cocetta G., Ferrante A., De Noni I., Piazza L., Morandi S. (2021). Assessment of possible application of an atmospheric pressure plasma jet for shelf life extension of fresh-cut salad. Foods.

[22] Ekesa B., Nabuuma D., Blomme G., Van den Bergh I. (2015). Provitamin A carotenoid content of unripe and ripe banana cultivars for potential adoption in eastern Africa. J. Food Compos. Anal..

[23] Spanos G.A., Wrolstad R.E. (1990). Influence of processing and storage on the phenolic composition of Thompson seedless grape juice. J. Agric. Food Chem..

[24] Re R., Pellegrini N., Proteggente A., Pannala A., Yang M., Rice-Evans C. (1999). Antioxidant activity applying an improved ABTS radical cation decolorization assay. Free Radic. Biol. Med..

[25] Boun H., Huxsoll C. (1991). Control of minimally processed carrot (*Daucus carota*) surface discoloration caused by abrasion peeling. J. Food Sci..

[26] Nishime T., Borges A., Koga-Ito C., Machida M., Hein L., Kostov K. (2017). Non-thermal atmospheric pressure plasma jet applied to inactivation of different microorganisms. Surf. Coat. Technol..

[27] Butscher D., Van Loon H., Waskow A., von Rohr P.R., Schuppler M. (2016). Plasma inactivation of microorganisms on sprout seeds in a dielectric barrier discharge. Int. J. Food Microbiol..

[28] Than H.A.Q., Nguyen T.T., Do N.K., Tran M.A.N., Pham T.H. (2025). Inactivation of *Diutina catenulata* isolated from Longan fruit using atmospheric pressure cold plasma DBD in argon, air, and argon-air mixture. Food Bioprocess Technol..

[29] Hertwig C., Leslie A., Meneses N., Reineke K., Rauh C., Schlüter O. (2017). Inactivation of Salmonella Enteritidis PT30 on the surface of unpeeled almonds by cold plasma. Innov. Food Sci. Emerg. Technol..

[30] Deng X., Shi J., Kong M.G. (2006). Physical mechanisms of inactivation of Bacillus subtilis spores using cold atmospheric plasmas. IEEE Trans. Plasma Sci..

[31] Laroussi M., Leipold F. (2004). Evaluation of the roles of reactive species, heat, and UV radiation in the inactivation of bacterial cells by air plasmas at atmospheric pressure. Int. J. Mass Spectrom..

[32] Georgescu N., Lungu C., Lupu A. (2010). Chemical activation of the high voltage pulsed, cold atmospheric plasma jets. Rom. Rep. Phys..

[33] Oehmigen K., Hähnel M., Brandenburg R., Wilke C., Weltmann K.D., Von Woedtke T. (2010). The role of acidification for antimicrobial activity of atmospheric pressure plasma in liquids. Plasma Process. Polym..

[34] Surowsky B., Froehling A., Gottschalk N., Schlueter O., Knorr D. (2014). Impact of cold plasma on *Citrobacter freundii* in apple juice: Inactivation kinetics and mechanisms. Int. J. Food Microbiol..

[35] Zhang B., Tan C., Zou F., Sun Y., Shang N., Wu W. (2022). Impacts of cold plasma technology on sensory, nutritional and safety quality of food: A review. Foods.

[36] Mei Y., Zhao Y., Yang J., Furr H. (2002). Using edible coating to enhance nutritional and sensory qualities of baby carrots. J. Food Sci..

[37] Yi F., Wang J., Xiang Y., Yun Z., Pan Y., Jiang Y., Zhang Z. (2022). Physiological and quality changes in fresh-cut mango fruit as influenced by cold plasma. Postharvest Biol. Technol..

[38] Baier M., Ehlbeck J., Knorr D., Herppich W.B., Schlüter O. (2015). Impact of plasma processed air (PPA) on quality parameters of fresh produce. Postharvest Biol. Technol..

[39] Kovačević D.B., Putnik P., Dragović-Uzelac V., Pedisić S., Jambrak A.R., Herceg Z. (2016). Effects of cold atmospheric gas phase plasma on anthocyanins and color in pomegranate juice. Food Chem..

[40] Lemmens L., Tibäck E., Svelander C., Smout C., Ahrné L., Langton M., Alminger M., Van Loey A., Hendrickx M. (2009). Thermal pretreatments of carrot pieces using different heating techniques: Effect on quality related aspects. Innov. Food Sci. Emerg. Technol..

[41] Ramazzina I., Berardinelli A., Rizzi F., Tappi S., Ragni L., Sacchetti G., Rocculi P. (2015). Effect of cold plasma treatment on physico-chemical parameters and antioxidant activity of minimally processed kiwifruit. Postharvest Biol. Technol..

[42] Santos Jr L., Cubas A., Moecke E., Ribeiro D., Amante E. (2018). Use of cold plasma to inactivate *Escherichia coli* and physicochemical evaluation in pumpkin puree. J. Food Prot..

[43] Fernandes F.A., Rodrigues S. (2021). Cold plasma processing on fruits and fruit juices: A review on the effects of plasma on nutritional quality. Processes.

[44] Bayati M., Lund M.N., Tiwari B.K., Poojary M.M. (2024). Chemical and physical changes induced by cold plasma treatment of foods: A critical review. Compr. Rev. Food Sci. Food Saf..

[45] Fernandes F.A., Santos V.O., Rodrigues S. (2019). Effects of glow plasma technology on some bioactive compounds of acerola juice. Food Res. Int..

[46] Bozkurt D., Kwiatkowski M., Terebun P., Diatczyk J., Pawłat J. (2018). Potential DBD-jet applications for preservation of nutritive compounds on the example of vitamin C in water solutions. Environmental Engineering V.

[47] Critzer F.J., Kelly-Wintenberg K., South S.L., Golden D.A. (2007). Atmospheric plasma inactivation of foodborne pathogens on fresh produce surfaces. J. Food Prot..

[48] Misra N., Roopesh M., Chemat F. (2019). Cold plasma for sustainable food production and processing. Green Food Processing Techniques.

[49] Kenny O., O’Beirne D. (2010). Antioxidant phytochemicals in fresh-cut carrot disks as affected by peeling method. Postharvest Biol. Technol..

[50] Simões A.D., Tudela J.A., Allende A., Puschmann R., Gil M.I. (2009). Edible coatings containing chitosan and moderate modified atmospheres maintain quality and enhance phytochemicals of carrot sticks. Postharvest Biol. Technol..

[51] Li M., Ren C., Li C., Fan Z., Zhu J., Qu C. (2025). Effect of Glow Discharge Cold Plasma Treatment on the Physicochemical Properties and Antioxidant Capacity of Maize. Foods.

[52] Reyes L.F., Villarreal J.E., Cisneros-Zevallos L. (2007). The increase in antioxidant capacity after wounding depends on the type of fruit or vegetable tissue. Food Chem..

[53] Heredia J.B., Cisneros-Zevallos L. (2009). The effect of exogenous ethylene and methyl jasmonate on pal activity, phenolic profiles and antioxidant capacity of carrots (*Daucus carota*) under different wounding intensities. Postharvest Biol. Technol..

[54] Li X., Li M., Ji N., Jin P., Zhang J., Zheng Y., Zhang X., Li F. (2019). Cold plasma treatment induces phenolic accumulation and enhances antioxidant activity in fresh-cut pitaya (*Hylocereus undatus*) fruit. LWT.

[55] Bagheri H., Abbaszadeh S. (2020). Effect of cold plasma on quality retention of fresh-cut produce. J. Food Qual..

[56] Zheng H., Miao T., Shi J., Tian M., Wang L., Geng X., Zhang Q. (2024). Effect of cold plasma treatment on the quality of fresh-cut hami melons during chilling storage. Horticulturae.

[57] Sudheesh C., Sunooj K.V. (2020). Cold plasma processing of fresh-cut fruits and vegetables. Fresh-Cut Fruits and Vegetables.

[58] Pour A.K., Khorram S., Ehsani A., Ostadrahimi A., Ghasempour Z. (2022). Atmospheric cold plasma effect on quality attributes of banana slices: Its potential use in blanching process. Innov. Food Sci. Emerg. Technol..

[59] Wu X., Zhao W., Zeng X., Zhang Q.-A., Gao G., Song S. (2021). Effects of cold plasma treatment on cherry quality during storage. Food Sci. Technol. Int..

[60] Zhang Y., Zhang J., Zhang Y., Hu H., Luo S., Zhang L., Zhou H., Li P. (2021). Effects of in-package atmospheric cold plasma treatment on the qualitative, metabolic and microbial stability of fresh-cut pears. J. Sci. Food Agric..

