# Supplementary material for: Microbial Decontamination of Fresh-Cut Carrots via Cold Atmospheric Plasma Treatment: Effect on Physicochemical and Nutritional Properties During Storage
Source: Foods. 2025 May 1;14(9):1599. doi: 10.3390/foods14091599 (PMC12071593; doi:10.3390/foods14091599)
Supplement: Supplementary file 1 [file foods-14-01599-s001.zip › foods-3550652-supplementary.pdf]

Supplementary File

Table S1. β-carotene levels, Ascorbic acid levels, total phenolic content and total antioxidant activity of plasma-treated and untreated (control) fresh-cut carrot samples during 3 weeks of refrigerated (4 °C) storage.

**Sample**	**Storage Period (week)**			
	**0**	**1**	**2**	**3**
	β-carotene (mg kg^{-1})			
**Control 1**	83.52±0.96 A	96.35±1.38 A	122.14±2.16 A	119.68±1.51 A
**Argon Plasma Treated**	81.39±1.17 A	94.04±0.79 B	118.81±1.18 A	116.33±1.85 B
**Control 2**	108.54±1.08 B	108.11±0.88 C	93.01±1.27 B	98.37±1.52 C
**Dry Air Plasma Treated**	103.46±1.16 C	102.66±1.33 D	88.24±0.94 C	93.23±1.17 D
	Ascorbic acid (mg kg-1)			
**Control 1**	49.29±0.20 A	46.38±0.19 A	34.82±0.34 A	43,00±0.59 A
**Argon Plasma Treated**	48.16±0.31 A	44.59±3.18 A	33.68±0.20 A	41.23±0.11 A
**Control 2**	40.50±0.30 B	51.93±6.65 B	37.91±0.27 B	49.03±1.09 B
**Dry Air Plasma Treated**	39.47±0.53 B	49.68±0.42 B	35.54±1.60 B	46.30±0.54 B
	Total Phenolic Content (GAE.100 g^{-1} fresh tissue)			
**Control 1**	64.70±0.73 A	70.30±1.90 A	76.42±1.42 A	72.45±1.09 A
**Argon Plasma Treated**	65.08±0.81 A	78.13±2,00 B	93.64±4.09 B	88.74±1.65 B
**Control 2**	69.84±0.40 B	72.83±0.82 A	76.91±1.77 A	78.98±1,01 C
**Dry Air Plasma Treated**	70.45±0.71 B	93.74±3.51 C	104.11±0.46 C	111.56±1.68 D
	Total Antioxidant Activity (mmol. L^{-1})			
**Control 1**	0.76±0.07 A	0.72±0.17 A	0.84±0.02 A	0.45±0.06 A
**Argon Plasma Treated**	0.73±0.14 A	0.79±0.03 A	0.99±0.14 B	0.56±0.03 B
**Control 2**	0.67±0.02 B	0.56±0.06 B	0.50±0.02 C	0.39±0.01 C
**Dry Air Plasma Treated**	0.64±0,00 B	0.63±0.12 B	0.64±0,05 D	0.51±0.16 B

Control 1: Control sample for dry air plasma treatment; Control 2: control sample for argon plasma treatment

Data are mean values ± standard deviation (n= 3). Values followed by different capital letters in the same column are significantly different ($p < 0.05$).
